# Protective Effects of Garlic-Derived S-Allylmercaptocysteine on IL-1*β*-Stimulated Chondrocytes by Regulation of MMPs/TIMP-1 Ratio and Type II Collagen Expression via Suppression of NF-*κ*B Pathway

**DOI:** 10.1155/2017/8686207

**Published:** 2017-12-03

**Authors:** Guang Yang, Siying Li, Bin Li, Lin Cheng, Peng Jiang, Zhoubin Tian, Shui Sun

**Affiliations:** ^1^Department of Joint Surgery, Shandong Provincial Hospital Affiliated to Shandong University, Jinan, Shandong 250021, China; ^2^Cheeloo College of Medicine, Shandong University, Jinan, Shandong 250000, China; ^3^Department of Orthopaedics, Jinan Central Hospital Affiliated to Shandong University, Jinan, Shandong 250013, China; ^4^Department of Endocrinology, Heze Municipal Hospital, Heze, Shandong 274000, China

## Abstract

**Background:**

Garlic-derived S-allylmercaptocysteine (SAMC) has widely been used in many disease therapies. However, the potential effects and mechanism of SAMC on IL-1*β*-stimulated chondrocytes are unclear.

**Methods:**

Chondrocytes were isolated, and 5 ng/mL of IL-1*β* was added to mimic the* in vitro* osteoarthritis (OA) model. SAMC (20 and 60 *μ*M) was used for the treatment in OA model. Cell viability was assessed by MTT method. Western blotting, Quantitative RT-PCR, and ELISA were performed to evaluate the mechanisms in SAMC treated OA model.

**Results:**

Following 48 h of IL-1*β* exposure, SAMC exhibited protection effect on IL-1*β*-injured chondrocyte viability. Type II collagen was elevated with reduced degradation products, as a consequence of altered MMPs/TIMP-1 ratio after SAMC treatment in IL-1*β*-treated chondrocytes. The protein and mRNA level of TNF-*α* in cellular supernatant and cells were downregulated in a dose-dependent manner. Besides, I*κ*B*α* in cytoplasmic fraction was increased, while p65 level in nuclear fraction was decreased after SAMC treatment in OA.

**Conclusions:**

This study showed that SAMC may play a protective role in IL-1*β* induced osteoarthritis (OA) model. This effect may be through inhibiting the NF-*κ*B signaling pathway, therefore altering the MMPs/TIMP-1 ratio change which induced type II collagen destruction and decreasing inflammatory cytokine secretion such as TNF-*α*.

## 1. Introduction

Osteoarthritis (OA) causes moderate to severe disability, which has a higher rate of morbidity in female than male, and pathogenic sites of OA are mainly finger joints, knee, hip, and spine [1]. OA is manifested as noninflammatory degeneration of articular cartilage and formation of joint marginal osteophyte, which usually results from aging and other reasons such as trauma, congenital anomaly, and deformity of joint [2, 3]. There are two kinds of OA treatment: nondrug therapy (health education, physical therapy, and professional treatment) and drug therapy (oral administration and intra-articular injections) [4]. However, the therapeutic outcomes are still unsatisfactory. For this reason, it is necessary to explore useful medicine or drugs for OA treatment.

Garlic and its derivatives have widely been used in many therapies, such as liver injury, renal damage, cardio-protection, cancer chemoprevention, and osteoarthritis [5]. Organosulfur compounds (OSCs) are the major active components in garlic, including the oil-soluble compounds, such as diallyl sulfide (DAS), diallyl disulfide (DADS), diallyl trisulfide (DATS), and water-soluble fractions containing S-allylcysteine (SAC) and S-allylmercaptocysteine (SAMC) [6]. Increasing evidence reports that garlic plays a pivotal role in relieving symptoms of OA patients in clinic [7, 8]. For instance, OSCs protect the injured chondrocytes through inhibiting nuclear factor (NF)-*κ*B activation [9]. Also, DAS downregulates COX-2 and PGE2 expression on joint inflammation induced by urate crystal and IL-1*β*, which suggests its potential protective role in OA treatments [10]. However, there are no reported studies devoted to explore the association of SAMC with application of OA treatment.

Type II collagen is the main component of cartilage matrix. Large portion of cartilage is reduced (worn away) over time with progression of osteoarthritis, making the type II collagen a specific biomarker for the breakdown of articular cartilage. The biomarkers detected in the synovial fluid, serum, and urine can achieve the early diagnosis and the development monitoring of osteoarthritis. Neoepitope of type II collagen C2C is a type II collagen proteolytic cleavage. In humans, serum and synovial fluid levels of C2C are suggested to be associated with OA progression [11].

Matrix metalloproteinases (MMPs) are essential mediators in extracellular matrix (ECM) destruction [12]. According to their main substrate specificity, MMPs can be divided into collagenases (MMP-1, MMP-8, and MMP-13), gelatinases (MMP-2 and MMP-9), stromelysins (MMP-3, MMP-10, and MMP-11), and membrane type I MMPs (MMP-14–17). Interestingly, MMP-9 involves in the breakdown of ECM in normal physiological processes such as tissue remodeling, as well as in disease processes, such as arthritis [13]. MMP-13 is expressed by chondrocytes and synovial cells in human OA and RA and is thought to play a critical role in cartilage destruction [14]. MMPs have been shown to participate in proteolytic cleavage of type II collagen [15]. So, MMPs-mediated cartilage degradation in OA generates the type II collagen fragments, which can be used as clinical biological markers. An important mechanism for the regulation of the activity of MMPs is via binding to a family of homologous proteins referred to as the tissue inhibitors of metalloproteinases (TIMPs) [16]. MMPs and their inhibitors have been identified as promising targets for OA treatment.

In this study, we isolated the chondrocytes from Sprague Dawley (SD) rat lumbar spines and constructed the OA model with the cytokine IL-1*β* stimulation [17]. And the potential role and mechanism of SAMC on OA were investigated.

## 2. Materials and Methods

### 2.1. Regents

SAMC (purity of 99%) was synthesized and purified in our laboratory with a modified procedure as previously reported [18]. SAMC was freshly prepared as a stock solution in PBS for the* in vitro* assay.

### 2.2. Cell Culture

The protocol of the animal experiments was performed in accordance with the institutional guidelines of the Animal Care and Use Committee of Shandong University. Chondrocytes isolation was conducted as previously described with modification [19]. Briefly, the SD rats weighted 300 ± 10 g at 12 weeks were killed with cervical dislocation for obtaining the lumbar spines within 1 h of death. Consequently, discs were carefully dissected using a microscope to collect the cartilage endplates. Tissues were digested with 0.25% trypsin (Sigma-Aldrich, St Louis, MO) for 2 h at 37°C, followed by maintaining in 0.02% collagenase (Sigma-Aldrich) for 24 h at 37°C. The enzymatic digested tissues were filtered through a 100 *μ*m cell strainer (BD Falcon, Palo Alto, CA) and then washed with phosphate buffered saline (PBS) buffer (PH 7.4) for 3 times. After that, the isolated cells were released from the matrix by centrifugation at 1,000*g* for 5 min at room temperature and then subjected onto 6-well plates at density of 2 × 10^4^ cells/well with the addition of Dulbecco's modified Eagle's medium (DMEM, Invitrogen, Carlsbad, CA) containing 10% fetal bovine serum (FBS, Invitrogen), 100 U/mL penicillin, and 100 *μ*g/mL streptomycin (Sigma-Aldrich) in an atmosphere of 5% CO_2_ at 37°C. After being cultured in high density monolayer culture for 1 week, isolated primary chondrocytes were trypsinized and then subjected onto 6-well plates for subsequent experiments.

### 2.3. OA Model and SAMC Treatment

Primary chondrocytes were divided into six groups: (1) Control: cells were not treated; (2) Model group: cells treated with 5 ng/mL of IL-1*β*; (3) SAMC alone: 60 *μ*M; (4) SAMC low dose group: cells treated with 5 ng/mL of IL-1*β* and 20 *μ*M of SAMC; (5) SAMC high dose group: cells treated with 5 ng/mL of IL-1*β* and 60 *μ*M of SAMC; (6) Dexamethasone group: cells were treated with 5 ng/mL of IL-1*β* and 10 ng/mL of dexamethasone. IL-1*β* (≥95%) and dexamethasone (≥97%) were both purchased from Sigma-Aldrich.

### 2.4. Cell Viability Assay

A stock solution of SAMC (5 mM) was prepared fresh in PBS. Cell viability was analyzed by the MTT (3-[4,5-dimethylthiazol-2-yl]-2,5 diphenyltetrazolium bromide) assay. Briefly, chondrocytes (5 × 10^3^) cultured in DMEM medium supplemented with 10% FBS at logarithmic phase were seeded on 96-well plates for adherence. Cells were treated with IL-1*β*, SAMC, and dexamethasone for 48 h, and then 10 *μ*L of MTT with final concentration of 5 mg/mL was added to the medium and the plates were incubated at 37°C for another 4 h. Supernatant was removed and then 150 *μ*L dimethyl sulfoxide (DMSO) was added to the cells for 10 min. Absorbance for cells in each well was read under an absorption spectrophotometer (AU600; Olympus, Tokyo, Japan) at 570 nm.

### 2.5. DAPI Staining

Chondrocytes were seeded into 24-well plates for 24 h. Then IL-1*β* and SAMC were directly added to the well and incubated for 48 h. The treated cells were washed with PBS and fixed with cold methanol/acetone (1 : 1, store at −20°C) for 5 min at room temperature. The solution was removed and washed with PBS and then incubated with DAPI solution for 10 min at room temperature. Fluorescent cells were observed under a fluorescence microscope (Olympus, Tokyo, Japan).

### 2.6. Western Blot Analysis

Total protein in chondrocytes was extracted by using RIPA lysis buffer (50 mM Tris (pH 7.4), 150 mM NaCl, 1% Triton X-100, 1% sodium deoxycholate, 0.1% SDS) containing protease and phosphatase inhibitor cocktails (Roche), at 4°C with vortex, and then was centrifuged at 300*g* for 10 min at 4°C. Proteins in the cytoplasm and nucleus were isolated by using Nuclear and Cytoplasmic Protein Extraction Kit (Beyotime, Shanghai, China), according to the manufacturer's instruction. The protein concentration was determined by BCA protein assay reagent (Pierce Biomedical Co., Rockford, IL). Cell lysates were separated using 10%–12% sodium dodecylsulfate-polyacrylamide gel electrophoresis (SDS-PAGE) and electro-transferred onto the polyvinylidene difluoride (PVDF) membrane (Mippore, Billerica, MA). The membranes were blocked in 5% nonfat milk for 1 h and then incubated with primary antibodies overnight at 4°C. The membranes were incubated with horseradish peroxidase labeled secondary antibody at room temperature for 1 h. The immunoreactive bands were visualized by an enhanced chemiluminescence reagent (Millipore) using Alphalmager HP system (Cell biosciences, USA).

The density of each band was measured using Image Pro Plus, standardized by the density of PCNA and *β*-actin. The primary antibodies included those of anti-Collagen II (ab34712, Abcam), anti-MMP9 (ab28898, Abcam), anti-MMP13 (ab39012, Abcam), anti-TIMP1 (ab61224, Abcam), anti-NF kappaB p65 (8242P, Cell signaling), anti-IкB-*α* (ab32518, Abcam), anti-PCNA (ab92552, Abcam), and *β*-actin (TA-09, ZSGB-BIO).

### 2.7. Enzyme-Linked Immune Sorbent Assay (ELISA)

TNF*α* concentration in culture supernatant was measured using the ELISA kit (Qiagen, Hilden, Germany). C2C concentration in culture supernatant was measured using the ELISA kit (eBioscience, US) according to the manufacture. CTX-II and Helix-II in culture supernatant were measured using Rat CTX-II (Cross Linked C-telopeptide of type II collagen) ELISA Kit and rat HELIX-II ELISA Kit (Westang, China). The culture supernatant was collected by centrifugation at 300*g* for 15 min for the measurement of released TNF*α* in chondrocytes in each group. Absorbance was read at 450 nm with a microplate reader (Bio-Rad, Hercules, CA).

### 2.8. Quantitative RT-PCR (qRT-PCR) Analysis

Total RNA from cells was extracted with Trizol extraction reagent (Qiagen), and then mixed DNA was removed with the addition of RNase-free Dnase I (Promega, Madison, WI). Concentration and purity of isolated RNA were measured with SMA4000 UV-VIS (Merinton, Shanghai, China). Purified RNA with concentration of 0.5 *μ*g/*μ*L was used for cDNA synthesis with PrimerScript 1st Strand cDNA Synthesis Kit (Invitrogen). Expressions of mRNAs were detected using SYBR green-based quantitative RT-PCR (Qiagen). Reaction systems of 20 *μ*L volume containing 1 *μ*L cDNA from the above PCR, 10 *μ*L SYBR Premix EX Taq, 1 *μ*L each of the primers (10 *μ*M), and 7 *μ*L ddH_2_O. Primers for target gene amplification were as follows: Tnf (NM_012675): 5′-CATTCCTGCTCGTGGCGGGG-3′, 5′-CGACGTGGGCTACGGGCTTG-3′; Gapdh (NM_008084.2): 5′-ATGTTCCAGTATGACTCCACTCACG-3′, 5′-GAAGACACCAGTAGACTCCACGACA-3′.

At the completion of cycling, melting curve analysis was performed to establish the specificity of the PCR product. The expression level of cDNA of each candidate gene was internally normalized using phosphoglyceraldehyde dehydrogenase (Gapdh). The relative quantitative value was expressed by the 2^−ΔΔCT^ method, representing the amount of candidate gene expression with the same calibrators. Each experiment was performed in triplicate and repeated three times.

### 2.9. Statistical Analysis

Data are expressed as mean ± standard derivation (SD) on the basis of at least three separate experiments. Statistical analysis was performed using one-way analysis of variance (ANOVA). Data in this study were calculated with the GraphPad Prism 5.0 (GraphPad Software Inc., San Diego, CA). *P* < 0.05 was considered as statistically significant.

## 3. Results

### 3.1. Effect of SAMC on Cells Viability and Apoptosis in IL-1*β*-Stimulated Chondrocytes

**Figure 1:**
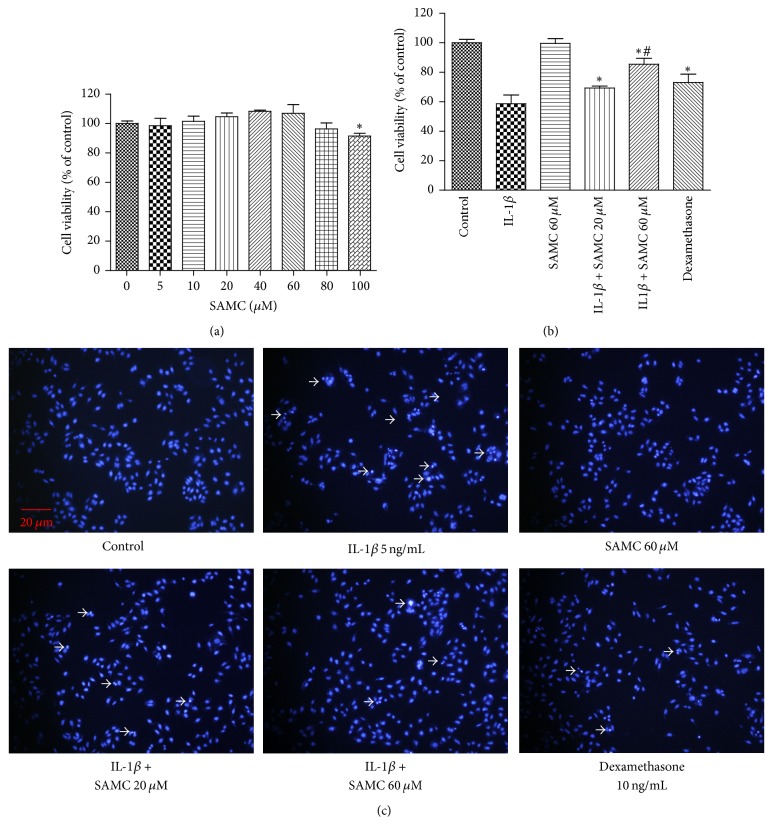
*Influence of SAMC on chondrocytes viability.* (a) Chondrocytes were treated SAMC with 0, 5, 10, 20, 40, 60, 80, and 100 *μ*M, and then cell viability was measured by MTT assay. (b) Chondrocytes were treated with IL-1*β*, SAMC alone (60 *μ*M), IL-1*β* plus SAMC (20 and 60 *μ*M), or IL-1*β* plus dexamethasone for 48 h, and then cell viability was measured. (c) DAPI staining of chondrocytes showed apoptotic body (arrow) after 48 h treatment. Data represent the mean ± SD of experiments in triplicate (Bars SD). ^*∗*^*P* < 0.05, versus IL-1*β* group; ^#^*P* < 0.05, versus dexamethasone group.

MTT assay was used to analyze the influence of SAMC on chondrocytes cell viability. As shown in Figure 1(a), there was no effect on cell viability or proliferation within the concentration range of 0 to 80 *μ*M for SAMC. After IL-1*β* treatment, cell viability was significantly inhibited at 48 h compared with control (Figure 1(b)), resulting in a viability decrease about 58.69%. Addition of SAMC and dexamethasone could increase cell viability, and SAMC (60 *μ*M) exhibited a much higher viability on cells than dexamethasone (85.40% versus 73.20%) (*P* < 0.05). Allicin, an organosulfur compound which is converted from alliin by alliinase of garlic, has been shown to participate in chondrocytes proliferation [20], so we examined the effect of SAMC in chondrocytes growth. The DAPI staining showed that, in IL-1*β*-treated chondrocytes, cells exhibited typical morphological signs of apoptosis, such as fragmented nuclei and apoptotic bodies, as evidenced by the arrows in Figure 1(c). The IL-1*β* induced cell apoptosis can be ameliorated by SAMC and dexamethasone.

### 3.2. Effect of SAMC on Type II Collagen Production and C2C Epitope Expression

**Figure 2:**
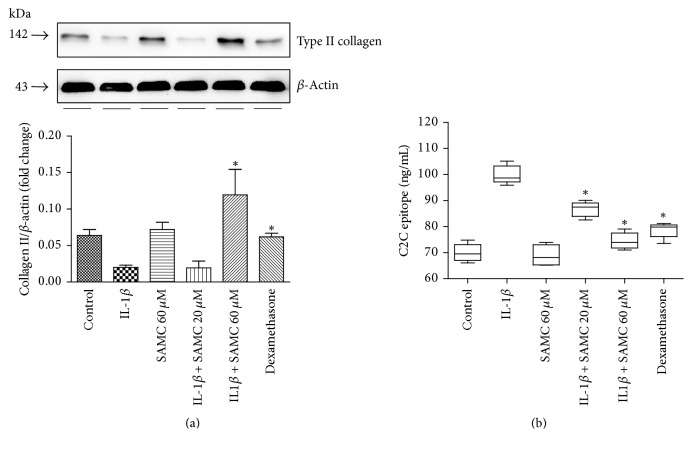
*Influence of SAMC on type II collagen and C2C expression in IL-1β-stimulated chondrocytes.* Chondrocytes were treated with IL-1*β*, SAMC alone (60 *μ*M), IL-1*β* plus SAMC (20 and 60 *μ*M), or IL-1*β* plus dexamethasone for 48 h. (a) The protein content of type II collagen was detected by Western blot. Protein expression levels were normalized to *β*-actin. (b) Level of type II collagen degradation protein C2C in the supernatant of cultured cells was detected by using ELISA. Data represent the mean ± SD of experiments in duplicate (Bars SD). ^*∗*^*P* < 0.05, versus IL-1*β* group.

Type II collagen is the basis for articular cartilage, making up 50% of all protein in cartilage and 85–90% of collagen, forming the fibrils to provide tensile strength to the tissue. Among several biomarkers reported, components of type II collagen are recognized as the most important biomarkers for OA [21]. Because type II collagen is specifically localized in cartilage and OA essentially involves catabolism and anabolism of articular type II collagen, we detected type II collagen expression in IL-1*β*-treated chondrocytes. As shown in Figure 2(a), level of type II collagen suppressed by IL-1*β* was restored by treatment with SAMC or dexamethasone, and SAMC (60 *μ*M) exhibited a more obvious effect. To be more specific to state the effect of SAMC in IL-1*β*-induced OA in rat chondrocytes, we made the test of collagen proteolytic products C2C in the supernatant of cultured cells by ELISA. On the contrary, the type II collagen cleaved products C2C expression was reduced after SAMC treatment in IL-1*β*-induced OA (Figure 2(b)).

### 3.3. Modulation of IL-1*β*-Induced MMPs and TIMP-1 Alteration by SAMC

**Figure 3:**
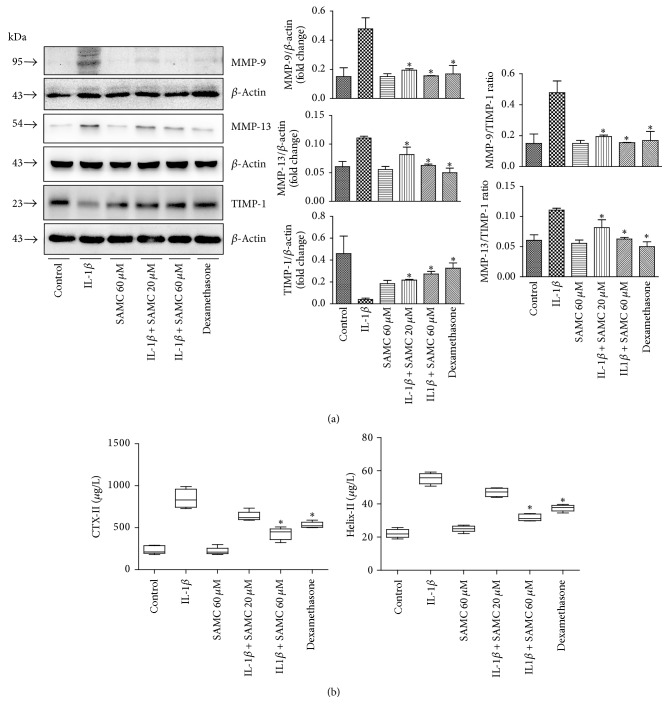
*Influence of SAMC on MMP-9, MMP-13, and TIMP-1 expression and MMP-mediated proteolytic cleavage of type II collagen in IL-1β-stimulated chondrocytes*. Chondrocytes were treated with IL-1*β*, SAMC alone (60 *μ*M), IL-1*β* plus SAMC (20 and 60 *μ*M), or IL-1*β* plus dexamethasone for 48 h. (a) The MMP-9, MMP-13, and TIMP-1 expressions were examined by western blot. (b) Type II collagen fragments, Helix-II and CTX-II, were measured using ELISA. Data represent the mean ± SD of experiments in duplicate (Bars SD). ^*∗*^*P* < 0.05, versus IL-1*β* group.

Altered MMP/TIMP-1 ratio is an important contributing factor in OA progression [22]. Effects of SAMC on MMP-9, MMP-13, and TIMP-1 protein expression in osteoarthritis chondrocytes were detected using western blot (Figure 3). The results showed that IL-1*β* significantly increased the protein expression of MMP-9 and MMP-13; on the contrary, TIMP-1 expression was significantly decreased compared with the control group. When adding SAMC and dexamethasone, MMP/TIMP-1 ratio was altered, indicating the inhibitory role of SAMC in IL-1*β*-induced OA.

### 3.4. Suppression of IL-1*β*-Induced NF-*κ*B Activation by SAMC

**Figure 4:**
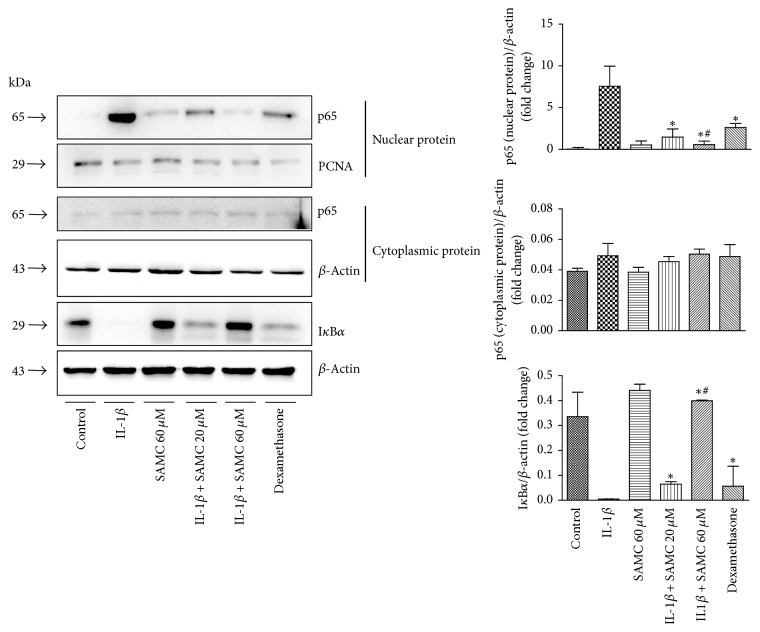
*Effects of SAMC on the NF-κB signaling in chondrocytes induced by IL-1β stimulation.* The protein expression of I*κ*B*α* in cytoplasmic fraction, p65 in nuclear and cytoplasmic fraction, was detected by western blot analysis. The *β*-actin acted as an internal control of I*κ*B*α* and cytoplasmic p65, and PCNA acted as an internal control of nuclear p65. Quantitative results of I*κ*B*α* and p65 levels based on western blotting. Data represent the mean ± SD of three independent experiments in duplicate (Bars SD). ^*∗*^*P* < 0.05, versus IL-1*β* group; ^#^*P* < 0.05, versus dexamethasone group.

To further explore the signaling pathway in IL-1*β*-induced arthritis, NF-*κ*B signaling pathway associated proteins were detected (Figure 4). The data showed that the I*κ*B*α* level in cytoplasmic fraction was decreased in IL-1*β*-stimulated chondrocytes, while the p65 level in nuclear fraction was increased, indicating that NF-*κ*B pathway was activated when chondrocytes were damaged by IL-1*β*. It seemed that SAMC and dexamethasone could block NF-*κ*B signaling (*P* < 0.05), as I*κ*B*α* in cytoplasmic fraction of these two groups was increased and p65 level in nuclear fraction was decreased in a dose-dependent manner.

### 3.5. Suppression of IL-1*β*-Induced TNF-*α* Elevation by SAMC

**Figure 5:**
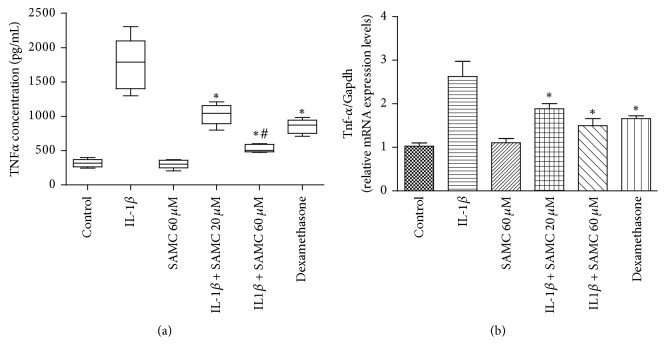
*Influence of SAMC on the expression of TNF-α*. Chondrocytes were treated with IL-1*β*, IL-1*β* plus SAMC, or IL-1*β* plus dexamethasone for 48 h. (a) The protein expression of TNF-*α* in cell supernatants was detected by ELISA assay. (b) Relative mRNA expression of Tnf-*α* in IL-1*β*-stimulated chondrocytes was measured by qRT-PCR analysis. Gapdh served as an internal control. Data represent the mean ± SD of three independent experiments in duplicate (Bars SD). ^*∗*^*P* < 0.05, versus IL-1*β* group; ^#^*P* < 0.05, versus dexamethasone group.

Furthermore, TNF-*α* level in cell culture supernatant and chondrocytes was also detected by ELISA and qRT-PCR. As the data given in Figure 5(a), TNF-*α* protein level in IL-1*β* treated group (1757.272 pg/mL) was much higher than control group (316.494 pg/mL). SAMC (20 and 60 *μ*M) could alleviate IL-1*β*-induced upregulation of TNF-*α* (*P* < 0.05) in a dose-dependent manner (1027.886 and 529.884 pg/mL, respectively). In Figure 5(b), TNF-*α* mRNA level in IL-1*β* treated group was elevated compared to in control group (2.63-fold versus 1.03-fold). SAMC (20 and 60 *μ*M) could alleviate IL-1*β*-induced upregulation of TNF-*α* (*P* < 0.05), respectively, resulting in a decrease of 1.88-fold and 1.50-fold.

## 4. Discussion

Previous studies have shown the correlation between garlic and OA treatment, but the effect of the major water-soluble fraction of garlic-derived S-allylmercaptocysteine (SAMC) still remains unclear. In this study, we constructed the OA model by treated the chondrocytes isolated from SD rats with IL-1*β* and analyzed the effects of SAMC on chondrocytes viability, type II collagen expression, MMPs/TIMP-1 ratio, and NF-*κ*B signaling pathway. The data presented in this study showed that SAMC increased total cell viability by preventing IL-1*β* induced cell apoptosis. Also, MMPs/TIMP-1 ratio was reversed by SAMC treatment with upregulation of type II collagen. Besides, SAMC significantly increased I*κ*B*α* in cytoplasmic fraction and decreased p65 level in nuclear fraction, a consequence of TNF-*α* downregulation.

IL-1*β* plays variety roles in OA, including promoting cartilage apoptosis, depredating cartilage matrix, and being involved in synovial inflammatory reactions [23]. It has been found with sharply abnormal level in synovial fluid from OA patients [24, 25]. Thus, in this study, IL-1*β* was used to induce chondrocyte injury to mimic an* in vitro* model of OA. As expected, 5 ng/mL of IL-1*β* significantly decreased chondrocyte viability and increased apoptotic cell body, which was consistent with previous studies [26].

Nowadays, several studies have focused on the effects of SAMC on cancer cells proliferation [27], but few focused on its role in OA. Li et al. have demonstrated that garlic and allicin presented protective effects on IL-1*β*-induced OA through increasing chondrocytes proliferation [20]. SAMC did not affect chondrocytes proliferation ability at basal status within 100 *μ*M. But our data showed that SAMC altered IL-1*β*-induced chondrocyte viability decreasing and reduced cell apoptosis, especially at 60 *μ*M concentration, suggesting the protective role of SAMC in IL-1*β*-induced chondrocyte death.

Type II collagen is the main component of cartilage matrix, which degraded in OA pathological progression [28]. In this study, we investigated the effects of SAMC on regulating the degradation of type II collagen. Importantly, IL-1*β* strikingly reduced the levels of type II collagen in chondrocytes, which was reversed by treatment with SAMC in a dose-dependent manner. In accordance with reduced degradation of type II collagen, its proteolytic cleavage products C2C had a decrease after SAMC treatment in the* in vitro* OA model.

Degradation of collagen in chondrocytes extracellular matrix components (ECM) is irreversible, and MMPs play a crucial part in depredating collagen in chondrocytes ECM and then enhance OA formation [29]. MMP-9 and MMP-13 protein levels were obviously higher in individuals with OA than those of normal people [13, 30]. In this study, upregulation of MMP-9 and MMP-13 was observed in IL-1*β*-stimulated chondrocytes, which is in accordance with antecedent studies [31]. More importantly, we found that SAMC could inhibit IL-1*β*-induced upregulation of MMP-9 and MMP-13. CTX-II and Helix-II are smaller type II collagen fragments, typically degraded by MMPs, which has been shown to provide a more accurate indication of the overall type II collagen degradation [32]. Indeed, MMP-13 appeared to be one of the most efficient collagenolytic enzymes. IL-1*β* induced more CTX-II and Helix-II production in chondrocytes, while SAMC reduced these collagen products production, which may be related to downregulation of MMPs, especially the collagenase MMP13. In addition, the biological activities of MMPs were antagonized by tissue inhibitors of metalloproteinases (TIMPs), such as TIMP-1 [33]. Thus, an imbalance in the activities of MMPs and TIMPs is considered to be critical in OA progression. A previous study has confirmed that IL-1*β* induces upregulation of MMPs and downregulation of TIMPs, resulting in degradation of proteoglycans and collagen [34]. The results of the present study revealed that SAMC significantly attenuated the IL-1*β* induced downregulation of TIMP-1, altered the MMPs/TIMP-1 ratio, and reduced type II collagen degradation, to protect against IL-1*β* induced OA in rat chondrocytes.

TNF-*α* is an important cytokine that is associated with pathogenesis of OA through involving in cartilage degradation and articular cartilage damage. Immunohistochemical detection showed that TNF-*α* level in OA tissue was abnormal compared with that in healthy persons, which suggested the potential correlation between TNF-*α* and OA pathogenesis [35]. In previous studies, SAMC has been demonstrated to inhibit cancer cell proliferation via JNK and p38 pathways [36] and promote cancer cell apoptosis by activating TGF-*β* and caspase 3 signaling [37, 38], furthermore, suppressing tumor growth through autophagy pathway [39]. Also, the NK-*κ*B signaling was mentioned in SAMC treated lung cancer cells [40], hepatocytes [41], and airway submucosal cells [42]. NF-*κ*B is an important intracellular transcription factor, involved in the pathophysiology of joint inflammation and tissue destruction [43]. It is well-established that IL-1*β*, once bound to its type 1 receptor, activates NF-*κ*B dimers by triggering phosphorylation and subsequent degradation of the inhibitory I*κ*B proteins. Activation of NF-*κ*B was a necessity for IL-1*β* induced MMP-13 secretion in OA chondrocytes [44, 45]. Also, Imamura et al. proved that IL-1*β* and TNF-*α* inhibited chondrogenesis through NF-*κ*B pathway in human mesenchyme stem cells [25]. Various garlic products have been studied in osteoarthritis [8]. And one sulfur compound isolated from garlic has demonstrated that it suppressed arthritis through inhibition of NF-*κ*B DNA-binding activity and expression of iNOS and COX-2 [9]. However, there are a few studies on SAMC effect on osteoarthritis. In addition, previous study has revealed that DATS suppressed MMP2-9 expression which was dependent on NF-*κ*B and ERK-MAPK Signaling Pathways [46]. Mechanistically, SAMC was found to be related to the increased levels of I*κ*B*α* induced by IL-1*β*, which subsequently mitigated p65 nuclear translocation and the transcriptional activity of NF-*κ*B. Furthermore, our results indicated that IL-1*β* treatment resulted in a significant increase in expression of the tumor necrosis factor (TNF-*α*) at both the mRNA and protein levels, which was ameliorated by treatment with SAMC. The combination of these findings suggests that SAMC can potentially be applied in OA treatment.

In conclusion, our study demonstrated that SAMC performed protective effect on resisting IL-1*β*-induced OA through reducing MMP-9 and MMP-13 with upregulated TIMP-1, thus protecting from type II collagen destruction* in vitro*. Mechanistically, TNF-*α* expression was downregulated with inhibiting NF-*κ*B pathway activation. Thus we concluded that SAMC may play a protective role in chondrocytes formation through suppressing TNF-*α* expression and inhibiting NF-*κ*B activation. However, it is not known how SAMC inhibits NF-*κ*B, in particular, what kind of surface receptors on chondrocytes bind with SAMC to transduce biological effects. Further studies that focus on the protective mechanism of SAMC on OA formation and progression* in vivo* are still needed.
